# Comprehensive Investigation of Electrical and Optical Characteristics of InGaN-Based Flip-Chip Micro-Light-Emitting Diodes

**DOI:** 10.3390/mi14010009

**Published:** 2022-12-21

**Authors:** Chang-Cheng Lee, Chun-Wei Huang, Po-Hsiang Liao, Yu-Hsin Huang, Ching-Liang Huang, Kuan-Heng Lin, Chung-Chih Wu

**Affiliations:** 1 Department of Electrical Engineering, Graduate Institute of Electronics Engineering, and Graduate Institute of Photonics and Optoelectronics, National Taiwan University, Taipei 106, Taiwan; 2 AU Optronics Corporation, Hsinchu 300, Taiwan

**Keywords:** flip-chip micro-LED, electrical modeling, optical modeling, light extraction, efficiency

## Abstract

Micro-light-emitting diodes (micro-LEDs) have been regarded as the important next-generation display technology, and a comprehensive and reliable modeling method for the design and optimization of characteristics of the micro-LED is of great use. In this work, by integrating the electrical simulation with the optical simulation, we conduct comprehensive simulation studies on electrical and optical/emission properties of real InGaN-based flip-chip micro-LED devices. The integrated simulation adopting the output of the electrical simulation (e.g., the non-uniform spontaneous emission distribution) as the input of the optical simulation (e.g., the emission source distribution) can provide more comprehensive and detailed characteristics and mechanisms of the micro-LED operation than the simulation by simply assuming a simple uniform emission source distribution. The simulated electrical and emission properties of the micro-LED were well corroborated by the measured properties, validating the effectiveness of the simulation. The reliable and practical modeling/simulation methodology reported here shall be useful to thoroughly investigate the physical mechanisms and operation of micro-LED devices.

## 1. Introduction

Ever since Jiang et al. delivered the first III-nitride blue micro-light-emitting diodes (micro-LEDs) in the year of 2000, research on micro-LED displays has become increasingly attractive and promising [1,2]. In contrast to liquid-crystal displays (LCD) and organic light-emitting-diode displays (OLEDs), micro-LED displays provide potential merits such as high brightness, high luminous efficiency, high dynamic range, long lifetime, short response time, low power consumption, and wide color gamut. The display applications of micro-LEDs could range from large high-performance TVs and mobile/portable devices to wearable devices for augmented reality (AR), virtual reality (VR), and smart watches. As a result, micro-LED displays have been now regarded as one of the important next-generation display technologies.

To develop high-performance micro-LEDs for displays, some issues and factors critical for micro-LED performance, such as: (i) current spreading, current crowding, and bulk/surface Shockley–Read–Hall recombination that would influence the carrier/emission distribution and internal quantum efficiency (IQE), and (ii) total internal reflection (TIR) loss inside the micro-LED chips that would affect the light extraction/luminous intensity/external quantum efficiency (EQE), require careful study and treatment. There have been some previous simulation studies investigating the physical mechanisms and operating behaviors of micro-LEDs [3,4,5,6]. Some works focused on the study and optimization of electrical properties [3,4], while some others paid attention to optical loss/light extraction properties [5,6]. Only a few studies considered/integrated both electrical and optical simulation of micro-LED chips simultaneously to give more complete and comprehensive views of micro-LED characteristics [7]. However, it is known that the current/carrier distributions and surface recombination phenomena inside the micro-LED mesa would influence the distribution of the spontaneous emission rate over the lateral active region and lead to an inhomogeneous emission distribution over the chip area. Nevertheless, many works have simply assumed a uniform emission distribution over the active region in the optical studies/simulation of micro-LEDs, which might lead to a deviation from the actual situations/characteristics (either chip-surface emission intensity distribution or far-field emission characteristics). Consequently, it would be more ideal and comprehensive to consider/integrate both the electrical and optical simulation of micro-LED chips simultaneously to obtain more a complete and comprehensive view and understanding of micro-LED characteristics.

In this work, we conduct comprehensive simulation studies, integrating the electrical simulation with the optical simulation, on real micro-LED devices and corroborate simulation results with experimental characteristics. The reliable and practical modeling/simulation methodology reported here shall be useful to thoroughly investigate the physical mechanisms and operation of micro-LED devices.

## 2. Research Methods

**Figure 1:**
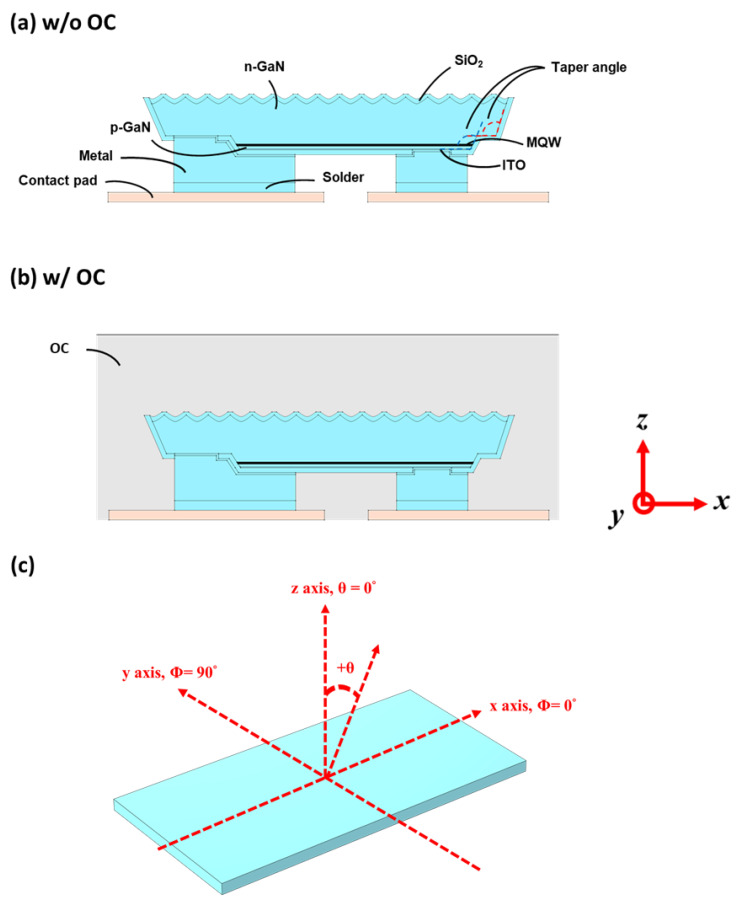
(**a**,**b**) The cross-sectional layer structure of the micro-LED chips transferred onto the substrate: (**a**) bare micro-LED without encapsulation overcoat (OC), and (**b**) micro-LED with encapsulation overcoat (OC). (**c**) The definition of the x-y-z coordinates, the polar angle *θ*, and the azimuthal angle *ϕ* relative to the long axis and short axis of the micro-LED chip.

The simulation studies were based on real fabricated blue GaN micro-LED devices/chips, as illustrated in Figure 1. As shown in Figure 1a, the rectangular flip-chip GaN micro-LEDs transferred and solder-bonded onto the substrate having metal interconnection bus electrodes had a size of 450 µm^2^ and a chip thickness of ~4.7–5 μm [5]. The sapphire substrates had been removed from the micro-LED chips by the lift-off process. The GaN micro-LEDs were grown on the pattered sapphire substrate (PSS), thus giving a textured chip surface after substrate lift-off (Figure 1a). Figure 1a further depicts the cross-sectional layer structure of the micro-LED chips. Epi-layers consisted of a ~0.3 μm p-GaN layer, a 0.02–0.05 μm p-AlGaN electron-blocking layer (p-EBL), ~0.2 μm InGaN multiple-quantum-well (MQW) active layers, and a ~4–5 μm n-GaN layer [3,8,9,10,11]. To expose the n-GaN for making n-contacts, a smaller mesa region containing the p-GaN/MQWs/partial n-GaN was formed by etching. The indium tin oxide (ITO) in contact with the p-GaN layer served as the current spreading layer for enhancing the electric conductivity and current spreading over the device [12]. The micro-LED chip was encapsulated with a thin SiO_2_ layer for passivation and for insulation to reduce electrical leakage [13]. Finally, the metal was applied (through openings in SiO_2_) as the p-contact to ITO/p-GaN and the n-contact to n-GaN [14]. The GaN micro-LED chips were transferred and solder-bonded onto the substrate having metal interconnection bus electrodes. The various sidewalls of the micro-LED chips had a taper angle of 110 ± 10° relative to the chip surface (Figure 1a) [5]. The PSS-induced surface texture had the concave shape of ~µm scale [15,16]. In addition to bare micro-LED chips bonded onto the substrate, even more practical/realistic micro-LED samples with a further encapsulation overcoat (OC, tens of micrometers thick over the micro-LED/substrate), as illustrated in Figure 1b, were also prepared and studied. Overall, two different micro-LED devices: (i) bare-surface-textured micro-LED without OC (Figure 1a), and (ii) surface-textured micro-LED with OC (Figure 1b), were analyzed in this study.

**Figure 2:**
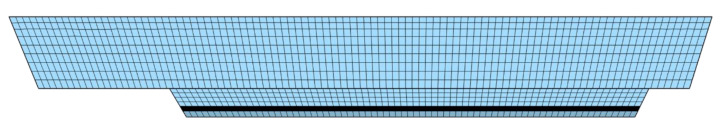
Illustration of the swept meshing method for numerical simulation of electrical characteristics of the micro-LED by the COMSOL Multiphysics^®^ software.

The electrical modeling/simulation was conducted using the semiconductor module of the FEM (finite element method)-based multiphysics simulation software COMSOL Multiphysics^®^ of COMSOL Inc., while the optical modeling/simulation was conducted using the Monte Carlo ray-tracing-based software LightTools^®^ of Synopsys Inc. The 3D electrical modeling/simulation of the micro-LED device was performed with the COMSOL semiconductor module, which solves device equations (Poisson’s equations, current discontinuity equation, carrier transport equation, and photon emission rate equation) based on the finite volume method. For most of the simulation, the operating temperature was mainly assumed at room temperature (300 °K). Considering large aspect ratios between the length, width, and height in the micro-LED layer stack and chip geometry studied, instead of using the default mesh geometry (tetrahedral mesh), it would be more effective to adopt the swept meshing technique (as illustrated in Figure 2) in the numerical simulation [10]. In the swept meshing, a boundary of quadrilateral meshes with a size of ~300 nm was first constructed as the source surface and the opposite targeted surface was specified. Then, an ample number of layers was set according to the thickness of the designated domain. Subsequently, the hexahedral elements were formed/arranged in order through the swept meshing. By means of the mesh optimization, the swept meshing technique not only reduces the number of meshes (compared with the default tetrahedral mesh), but also enables numerical solutions to reach convergence more effectively (e.g., with reasonable/reduced computing resource and time).

In reference to material growth conditions, Table 1 lists the material properties of various micro-LED layers used for electrical modeling/simulation [17,18,19,20,21,22,23,24]. The material layers were assumed as heavily doped near the electrode to ensure ohmic contacts to electrodes. To analyze the dependence of internal quantum efficiency (IQE) on the current density, non-ideal non-radiative recombination factors such as Shockley–Read–Hall recombination (both the bulk one and the trap-assisted surface recombination) and Auger recombination were considered in the numerical simulation. IQE $\eta_{IQE}$ can conceptually be expressed as [3]:

(1) $$\eta_{IQE}=\frac{R_{Rad}}{R_{Rad}+R_{Auger}+R_{SRH}},\;$$

where *R_rad_* is the radiative recombination rate, *R_Auger_* is the Auger recombination rate, and *R_SRH_* is the (bulk or surface) Shockley–Read–Hall recombination rate. Assuming the electron and hole concentrations are much larger than the intrinsic carrier concentrations, the Auger recombination rate *R_Auger_* and Shockley–Read–Hall recombination rate *R_SRH_* can be expressed as [4]:

(2) $$R_{Auger}=(C_{n}n+C_{p}p)np$$

(3) $$R_{SRH}=\frac{np}{\tau_{n}n+\tau_{p}p}\;(\mathrm{bulk}),\;R_{SRH}=\frac{np}{\frac{n}{v_{s,p}}+\frac{p}{v_{s,n}}}\;(\mathrm{surface})$$

*n* and *p* are electron and hole concentrations, *C_n_* and *C_p_* are the auger recombination coefficients, *τ_n_* and *τ_p_* are electron and hole lifetimes (in the bulk) for the Shockley–Read–Hall recombination, and *v_s,n_* and *v_s,p_* are the surface recombination velocities (SRVs) for both electrons and holes, respectively. In general, *R_Auger_* is more significant at the high current densities, while *R_SRH_* is dominant at the low current densities (thus, under general operating conditions of micro-LED devices). In view of the high perimeter-to-area ratios of micro-LEDs, the trap-assisted surface non-radiative recombination occurring at mesa surfaces was also considered [3], setting the trap levels 0.46 eV above the valence band and 0.24 eV below the conduction band [25], and setting the surface recombination velocities (SRVs) for both electrons and holes (*v_s,n_* and *v_s,p_*, respectively) around 1000 cm/s (Table 1) [13]. Through electrical simulation under different biases, the spatial distributions of carrier concentrations, current flows, various recombination rates, and spontaneous emission rates as a function of bias, J–V characteristics, and electroluminescence (EL) and IQE as a function of bias can be analyzed. From the electrical modeling/simulation, the (three-dimensional) distributions of current flows, carriers, recombination, and spontaneous emission in the micro-LED device under different biases can be visualized.

With the spatial (non-uniform) distribution of radiative recombination rates (spontaneous emission rates) calculated from the electrical modeling/simulation by COMSOL, the data were transformed into a matrix corresponding to locations over the MQW active region and were then used to set-up the spatial light source distributions over the MQW active region for optical modeling/simulation in LightTools. At each location, the s-polarized and p-polarized light sources were established separately with isotropic and cos^2^θ angular distributions and the total radiation power ratio of 3:1 (s vs. p polarization), respectively [26]. In addition, the total radiation powers (sum of s and p polarizations) and, thus, the total light ray numbers over locations were determined by the spatial distribution of the spontaneous rates over the active region calculated by the COMSOL. Overall, the effects of polarization, angular distribution, and spatial distribution were all taken into account in our optical simulation model. As the optical microcavity effects in the micro-LED configuration had been reported to be weak and relevant micro-LED dimensions (sizes, thicknesses, etc.) were larger than emission wavelengths [5], the optical modeling/simulation was conducted by the 3D Monte Carlo ray-tracing method, using the Synopsys LightTools software. Table 2 lists refractive indices and extinction coefficients at the emission wavelength of 459 nm for various materials/layers used in the optical simulation [5]. The small but not negligible extinction coefficient of the MQW layers would induce absorption loss upon propagation and multiple reflections of light. Tens of millions of rays propagating from the MQW emission region through multilayers and interfaces were calculated according to Snell’s law and Fresnel equations. A planar receiver was placed 20 nm above the micro-LED sample surface to detect the normal-direction surface emission intensity distribution in correspondence with the measurement setup, while a large (infinite) sphere far-field receiver was used to observe/collect the far-field emission characteristics such as the light out-coupling/extraction efficiencies and emission patterns. The emission patterns were represented by the emission intensity as a function of the polar angle *θ* under various azimuthal angles *ϕ*, in which *ϕ* = 0° and *ϕ* = 90° correspond to the long axis and short axis of the micro-LED chip (see Figure 1c), respectively. The external quantum efficiency (EQE) $\eta_{EQE}$ in general can be expressed as:

(4) $$\eta_{EQE}=\eta_{CIE}\times \eta_{IQE}\times \eta_{LEE},$$

where $\eta_{CIE}$ is current injection efficiency, $\eta_{IQE}$ is internal quantum efficiency, and $\eta_{LEE}$ is light extraction efficiency [3]. For verification of optical simulation, simulation results were compared with the experimental measurements. The current-density–voltage (J–V) characteristics of the devices were measured by a source-measurement unit (Keithley 2400 SourceMeter, Tektronix Inc., Beaverton, OR, USA). The external quantum efficiencies (EQEs) of devices were determined by collecting the total emission fluxes with a calibrated spectral lamp measurement system (model SLM-12, AMA Optoelectronics Inc., Taoyuan, Taiwan), including a 12″ integrating sphere, a spectrometer (VNIR1010, Isuzu Optics Corp., Hsinchu, Taiwan), and a source meter (Agilent E3632A, Agilent Technologies Inc., Santa Clara, CA, USA). Angle-dependent EL properties (e.g., far-field emission patterns) of the devices were measured by a goniometric spectroradiometer (DMS 201, Autronic-Melchers GmbH, Karlsruhe, Germany). The emission intensity distributions over the micro-LED sample surface were observed using an optical microscope (MX50, Olympus Corporation, Tokyo, Japan) equipped with a CMOS sensor (SR-5100, Topcon corporation, Tokyo, Japan).

## 3. Results and Discussion

**Figure 3:**
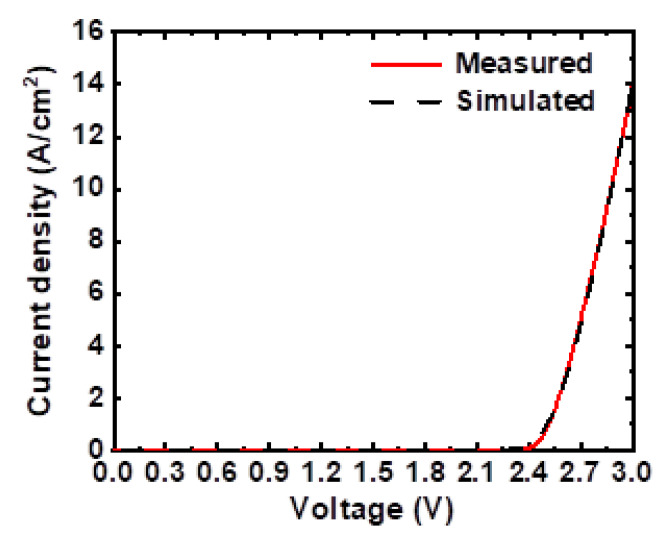
Calculated and measured current-density–voltage (J–V) characteristics of the micro-LED.

By the numerical simulation approaches described in the previous section, various electrical and physical characteristics for the micro-LED chip were calculated and analyzed. Figure 3 shows simulated current-density–voltage (J–V) characteristics, which are in satisfactory agreement with the measured ones. It is worth mentioning that the operating current densities for display applications of micro-LEDs are usually in the range of 0.02 to 2 A/cm^2^ [3], substantially lower than those of conventional large-size LED applications (e.g., lighting).

**Figure 4:**
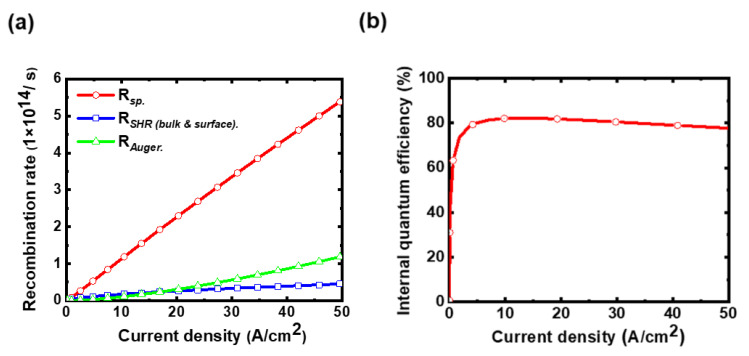
(**a**) The calculated spontaneous emission rate and non-radiative recombination rates vs. current density. (**b**) The calculated IQE vs. current density.

**Figure 5:**
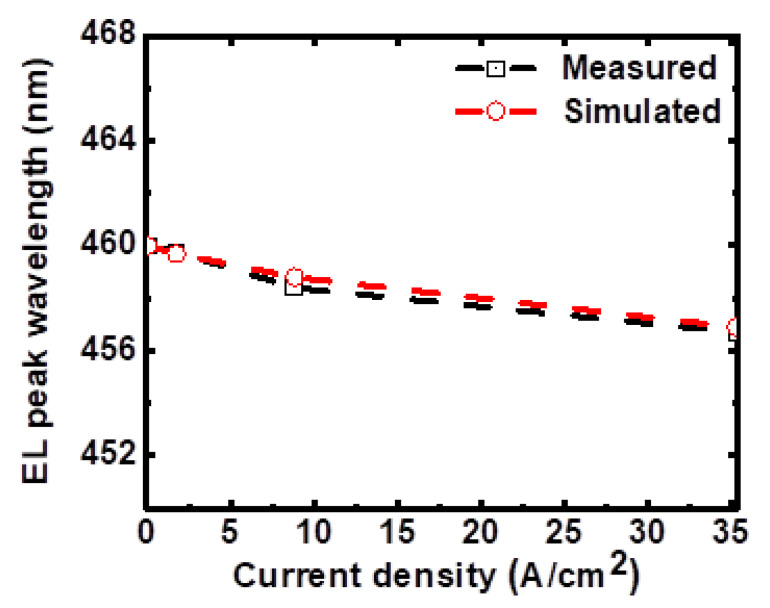
Calculated and measured EL peak wavelength as a function of the current density.

Figure 4a shows the calculated volume-integrated spontaneous emission rate (*R_sp._*) and non-radiative recombination rates (*R_SRH_*, *R_Auger_*) vs. current density characteristics of the micro-LED. It is seen that *R_SRH_* is comparable to *R_sp._* or even more dominant at the low current densities, while *R_Auger_* becomes more significant at higher current densities. Correspondingly, in the calculated IQE vs. current density characteristics in Figure 4b, the drop in IQE at lower current densities is associated with more significant influences of *R_SRH_*, while the slight IQE roll-off at higher current densities is due to gradually more significant *R_Auger_* [27]. The peak IQE (~82.3%) occurs around the medium current density of 12.8 A/cm^2^. The micro-LED device under study exhibits a peak IQE at the (relatively low) current density level useful for display applications, which would be beneficial for the overall EQE of the micro-LED displays. The calculated electroluminescence (EL) peak wavelengths under different current densities are shown in Figure 5. A blue shift of ~3.1 nm in the peak wavelength is observed with increasing current density from 0.044 to 35.3 A/cm^2^, as often observed and caused by the piezoelectric-induced quantum-confined Stark effect (QCSE) in the MQW layer [28]. This simulation result again is in good agreement with measured values in experiments (also shown in Figure 5).

**Figure 6:**
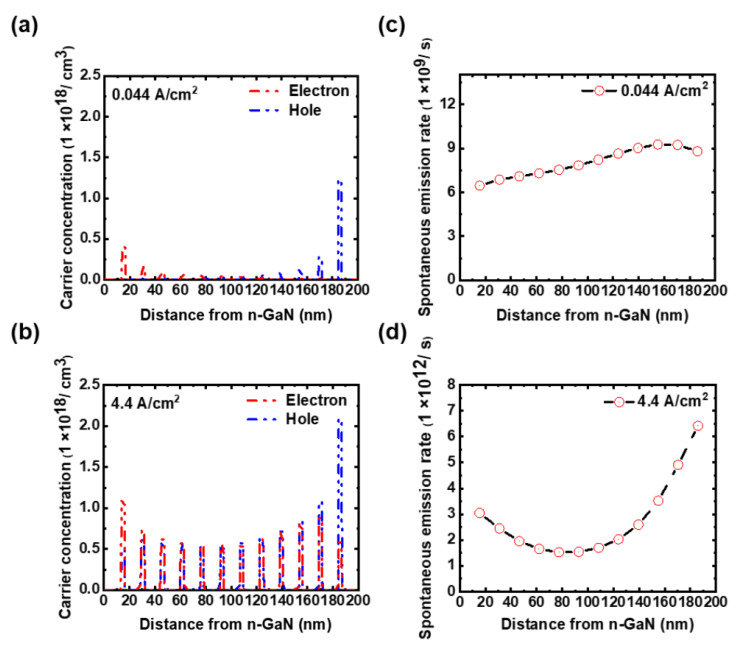
(**a**,**b**) The spatial distributions of electron and hole concentrations in the MQW active region (on top of the p-contact center) at the current densities of 0.044 A/cm^2^ and 4.4 A/cm^2^; (**c**,**d**) the spatial distributions of the total spontaneous emission rates of individual QWs in the MQW active region under the current densities of 0.044 A/cm^2^ and 4.4 A/cm^2^.

To gain more insights into the operation of the MQW-based micro-LEDs, Figure 6a,c and Figure 6b,d show the spatial distributions of carrier concentrations (both electrons and holes) and total spontaneous emission rates of individual QWs in the MQW active region under the operating current densities of 0.044 A/cm^2^ and 4.4 A/cm^2^, respectively. With large effective mass and low mobility for holes, the hole concentration decreases from the QW adjacent to the p-side to that close to the n-side, while additional high-electron concentrations could occur in QWs near the p-side owing to the electron-blocking layer. As both electrons and holes have high carrier concentrations in QWs near the p-side (Figure 6a,b), higher spontaneous emission rates occur in QWs near the p-side (Figure 6c,d).

**Figure 7:**
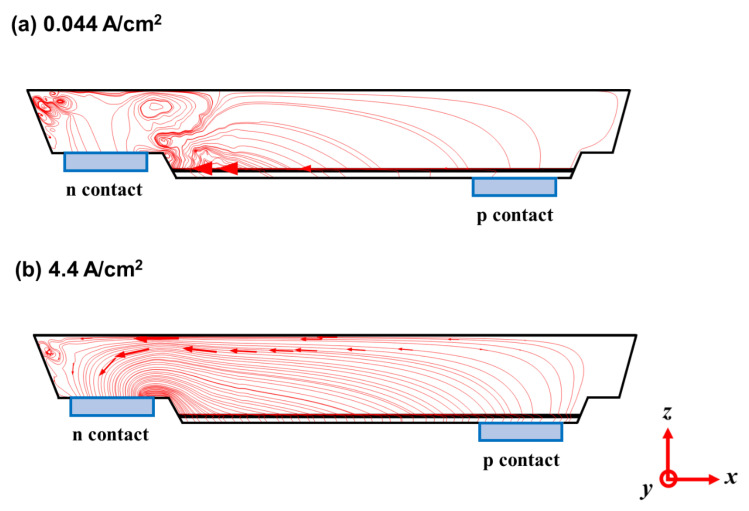
The current streamlines inside the micro-LED chip under the operating current densities of (**a**) 0.044 A/cm^2^ and (**b**) 4.4 A/cm^2^.

To gain more insights into the distribution of current flows and current paths, the current streamlines inside the micro-LED under different operating current densities were also calculated and are visualized in Figure 7a (0.044 A/cm^2^) and Figure 7b (4.4 A/cm^2^). At the low current density (0.044 A/cm^2^), the somewhat current crowding near the MQW mesa edge next to the metal n-contact is observable. It indicates that the ITO film is relatively conductive (compared to other GaN-based layers) for laterally conducting currents (injected from the metal p-contact) at low current densities, thus being the important factor influencing the current streamlines at low current densities [29]. On the other hand, at higher current densities, more uniform current spreading from the edge of the metal p-contact toward the metal n-contact is noticeable. This is presumably because the carrier concentration and the corresponding conductivity of the n-GaN region are substantially enhanced at higher current densities, making it now effective for laterally conducting currents. It, thus, could be deduced that the raised conductivity of n-GaN is the important factor to affect the current flowing paths at higher current density levels.

**Figure 8:**
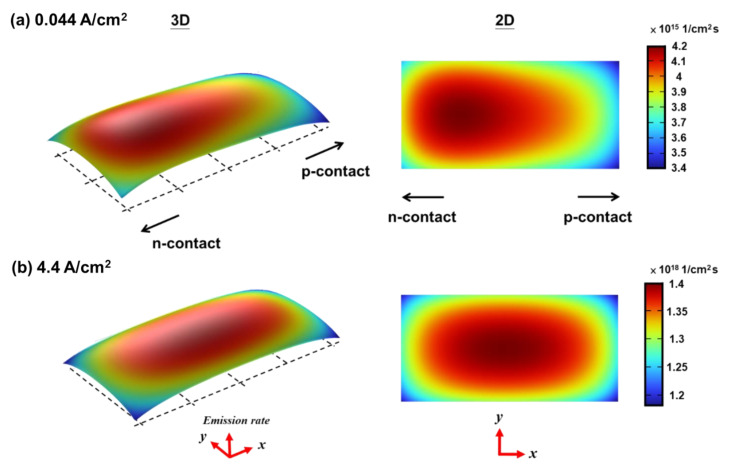
The non-uniform distribution of spontaneous emission in the active region under the operating current densities of (**a**) 0.044 A/cm^2^ and (**b**) 4.4 A/cm^2^.

Figure 8a,b further show the distributions of the spontaneous emission rates in both 3D and 2D formats over the MQW active area at the low current density (0.044 A/cm^2^) and at the higher current density (4.4 A/cm^2^), respectively. They generally reveal a somewhat non-uniform spontaneous emission distribution. More intense emission occurs around the edge of the metal n-contact (Figure 8a) due to somewhat current crowding at low current densities (Figure 7a). On the other hand, more intense spontaneous emission shifts toward the center of the MQWs at higher current densities (Figure 8b) due to more uniform current spreading (Figure 7b). In addition, the intensity of the spontaneous emission drops near the MQW mesa edges, which could be ascribed to current leakage and non-radiative surface recombination on mesa sidewall surfaces [30]. In particular, such a phenomenon is relatively more significant at low current densities [3].

**Figure 9:**
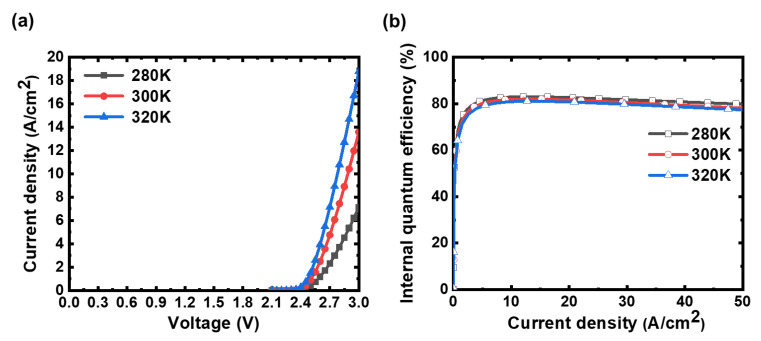
(**a**) The calculated temperature-dependent current-density–voltage (J–V) characteristics; (**b**) the calculated temperature-dependent IQE vs. current density.

To gain more insights into the real operation of micro-LEDs, the influences of temperatures on electrical properties are also simulated and discussed. Figure 9a shows the simulated temperature-dependent current-density–voltage (J–V) characteristics. The current density increases with rising temperature, which is consistent with previous studies and can be ascribed to band-gap narrowing and increased carrier concentration in the active region [31,32,33]. Figure 9b depicts the calculated temperature dependence of IQE vs. current density characteristics. The peak IQE decreases slightly from 83% to 81% when raising the temperature from 280 K to 320 K, mainly associated with the temperature dependence of radiative and non-radiative recombination rates. The Shockley–Read–Hall and Auger recombination ascend while the radiative recombination descends as the temperature increases for the MQWs according to previous studies [33,34]. Further detailed analyses also reveal that the spatial distribution of the spontaneous rate would be influenced by the temperature. The more intense spontaneous emission shifts more toward the n-contact at elevated temperatures due to the variation in carrier and current distributions.

**Figure 10:**
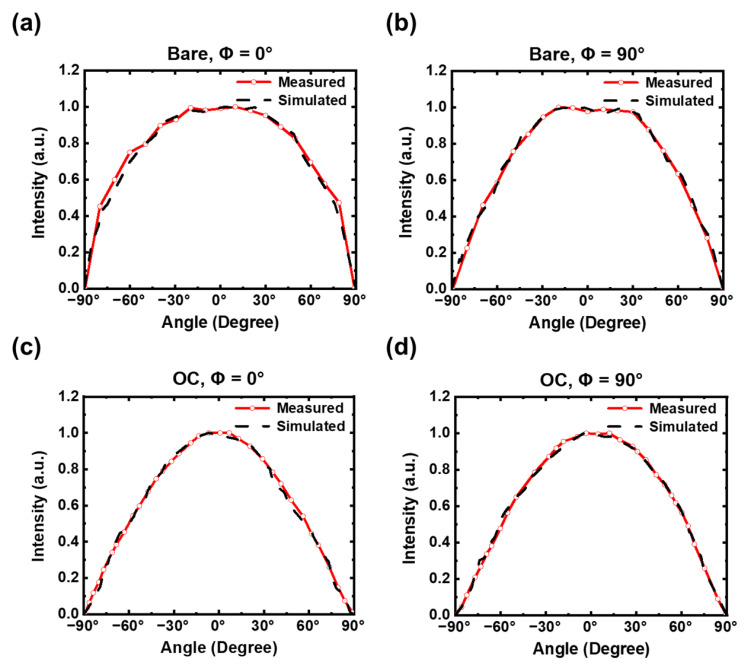
Simulated far-field emission patterns (emission intensity as a function of the polar angle *θ*) of the bare micro-LED (i.e., no OC) and the OC-encapsulated micro-LED along the chip long axis (*ϕ* = 0°) and along the short axis (*ϕ* = 90°), in comparison with corresponding measured emission patterns (under the current density of 4.4 A/cm^2^). Bare, *ϕ* = 0° (**a**). Bare, *ϕ* = 90° (**b**). OC, *ϕ* = 0° (**c**). OC, *ϕ* = 90° (**d**).

As described in the previous method section, we then used the simulation results of the electrical modeling/simulation of the micro-LED (e.g., distributions of spontaneous emission rate/intensity as in Figure 8) as inputs to set-up the emission sources in the active region of the micro-LED, together with optical constants of various material layers listed in Table 2, for further optical modeling/simulation. Figure 10 shows the simulated far-field emission patterns (emission intensity as a function of the polar angle *θ*) of the bare micro-LED (i.e., no OC) and the OC-encapsulated micro-LED (i.e., those in Figure 1a,b) along the chip long axis (*ϕ* = 0°) and along the short axis (*ϕ* = 90°), in comparison with corresponding measured emission patterns. The good agreement between the optical simulation results and the measurement results indicates effectiveness of the overall modeling/simulation methodology here (integrated electrical and optical simulation). Indeed, the optical simulation can quite accurately predict the unsymmetric/different emission patterns along the long axis (*ϕ* = 0°) or the short axis (*ϕ* = 90°), associated with unsymmetric chip geometry. It also well predicts the narrowing of emission patterns induced by the encapsulation overcoat (OC). The narrowing of emission patterns with the OC is mainly associated with the total internal reflection (TIR) at the OC–air interface. For light rays coupled into the OC with an initial internal angle larger than the TIR, the critical angle of the OC–air interface would be confined inside the OC, while for the bare micro-LED, more large-angle light rays extracted into air from various surfaces (including the perimeter sidewalls) can be directly observed.

The bare surface-textured micro-LED bonded onto the substrate gives a light extraction efficiency *η_LEE_* of 40.8% at 4.4 A/cm^2^, which is roughly independent of the driving current density (not shown). With the encapsulation overcoat (OC), the light extraction efficiency drops significantly to 16.4% (a reduction by nearly 60%). A similar efficiency drop is also observed in the EQE measurement at the same current density (i.e., ~60% efficiency drop). The consistency between efficiency simulation and measurement for different device configurations again confirms the effectiveness of the simulation. According to detailed analyses, the coupling ratio of micro-LED emission into the OC layer (~70%) is significantly higher than that directly into air (40.8%) from the bare micro-LED due to the higher index (~1.5) of the OC layer. However, most of the emission coupled into the thick OC layer is confined (by total internal reflection at the OC/air interface) in the thick OC layer, resulting in reduced overall $\eta_{LEE}$ for the OC-encapsulated micro-LED (vs. bare micro-LED).

**Figure 11:**
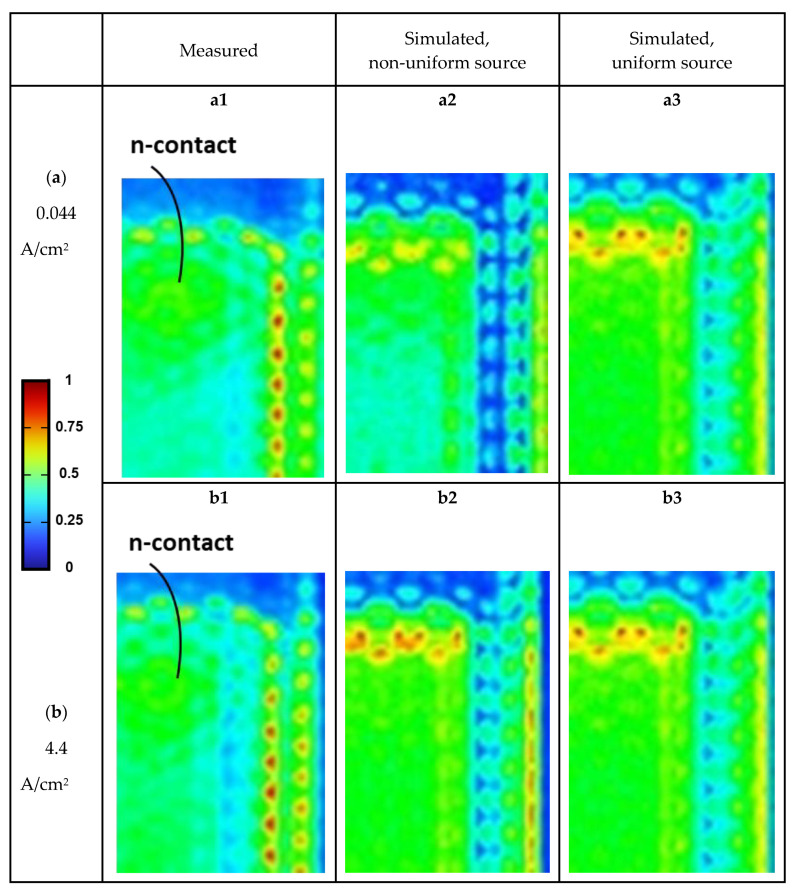
Measured and simulated (normalized) emission intensity distributions of the surface-textured and OC-encapsulated micro-LED under the low driving current density of 0.044 A/cm^2^ and the higher driving current density of 4.4 A/cm^2^. (**a1**,**b1**) Measured profiles. (**a2**,**b2**) Simulated profiles, simulated using inhomogeneous emission source distributions calculated by the electrical stimulation. (**a3**,**b3**) Simulated profiles, simulated using simple uniform emission source distributions.

Figure 11(a1,b1) show the measured (normalized) emission intensity distributions over a portion of the sample surface of the OC-encapsulated micro-LED under the low driving current density of 0.044 A/cm^2^ and the higher driving current density of 4.4 A/cm^2^, respectively. In measured emission intensity distribution profiles, several distinct emission features can be observed: (i) distinct emission around the chip edges/perimeter (outside the MQW mesa area) and the MQW mesa edges/perimeter is observed, which can be ascribed to the light extraction effect of the tapered chip/MQW mesa sidewalls (i.e., re-direction and out-coupling of confined and laterally propagating light into the front-side emission by the tapered sidewalls); (ii) stronger emission from the overlap areas of the MQW mesa and the contact electrodes, due to optically reflective characteristics of the metal electrodes; (iii) consistent with calculated distributions of the spontaneous emission rates over the MQW active area shown in Figure 8, relatively weaker emission is seen around the center of the MQW mesa at the low current density (0.044 A/cm^2^) due to the current crowding/larger emission rate near the n-contact edge, while relatively stronger and more uniform emission is seen around the center of the MQW mesa at the higher current density (4.4 A/cm^2^) due to more uniform current spreading and a shift in more intense spontaneous emission toward the mesa center; (iv) also consistent with calculated distributions of the spontaneous emission rates in Figure 8, weaker-emission mesa areas appear near the MQW mesa edges, presumably associated with non-radiative surface recombination on mesa sidewalls.

These measured profiles are compared with simulated emission intensity distribution profiles, simulated using inhomogeneous emission source distributions (as shown in Figure 8) calculated by the electrical modeling/simulation (Figure 11(a2,b2)), or using simple, conventional uniform emission source distributions (Figure 11(a3,b3)). Both simulation approaches can more or less predict or reproduce optical effects induced by the chip geometries and material optical properties (e.g., re-direction out-coupling by tapered sidewalls, surface textures, and reflection by the metal electrodes). Yet, the simple optical model adopting the uniform emission source distribution fails to simulate some non-uniform emission characteristics over the MQW mesa area (e.g., those associated with different driving current density or non-radiative surface recombination), while the model adopting the emission source distribution input from the electrical modeling/simulation can better reproduce such characteristics. For instance, the optical model with the electrical simulation input can simulate weaker emission around the center of the MQW mesa at the low current density and stronger/more uniform emission around the center of the MQW mesa at the higher current density. Such results clearly indicate that the integration of both electrical and optical simulation is useful to provide more detailed and comprehensive characteristics and mechanisms of the micro-LED operation.

## 4. Conclusions

In this work, by integrating the electrical simulation with the optical simulation, we conducted comprehensive simulation studies on electrical and optical/emission properties of real InGaN-based flip-chip micro-LED devices. Through the electrical modeling/simulation, electrical properties, such as J–V characteristics, carrier, current, and spontaneous emission rate distributions, and internal quantum efficiencies, were first calculated. The output of the electrical simulation (e.g., the non-uniform spontaneous emission distribution) was then adopted as the input of the optical simulation (e.g., the emission source distribution) for further calculating optical/emission properties, such as light extraction efficiency, external quantum efficiency, and far-field and intensity distribution profiles. It was found that such an integrated simulation approach can provide more comprehensive and detailed characteristics and mechanisms of the micro-LED operation than the optical simulation by simply assuming a simple uniform emission source distribution. The simulated electrical and emission properties of the micro-LED were well corroborated by corresponding measured properties, validating the effectiveness of the overall simulation methodology. The reliable and practical modeling/simulation methodology reported here, thus, shall be useful to thoroughly investigate the physical mechanisms and operation of micro-LED devices.

## Figures and Tables

**Table 1:** Materials properties of various micro-LED layers used for electrical modeling/simulation.

Material Layer	Material Properties
n^+^-GaN	Doping: 2 × 10^18^ 1/cm^3^
n-GaN	Doping: 1 × 10^18^ 1/cm^3^ [17]
MQW	Non-doped ^(a)^ *τ_sp_* = 1 ns ^(b)^ *C_n_*, *C_p_* = 8 × 10^−31^ cm^6^/s ^(c)^ *τ_n_*, *τ_p_* = 200 ns ^(d)^ *v_s,n_*, *v_s,p_* = 1000 cm/s
AlGaN	Doping: 3 × 10^18^ 1/cm^3^ [3]
p-GaN	Doping: 3 × 10^18^ 1/cm^3^ [21]
p^+^-GaN	Doping: 8 × 10^18^ 1/cm^3^
p^+^-GaN/ITO contact	Specific resistance: 1.7 × 10^−2^ Ω-cm [22]
ITO	Resistivity: 4 × 10^−4^ Ω [23]
ITO/metal contact	Specific resistance: 3 × 10^−4^ Ω-cm [24]

^(a)^ *τ_sp_* is the spontaneous lifetime of the emission layer; ^(b)^ *C_n_, C_p_* are the auger recombination coefficients for both electrons and holes, respectively, whose values are taken from reference [18]; ^(c)^ *τ_n_, τ_p_* are, respectively, electron and hole lifetimes (in the bulk) for the Shockley–Read–Hall recombination, whose values are taken from reference [19]; ^(d)^ *v_s,n,_ v_s,p_* are the surface recombination velocities (SRVs) for both electrons and holes, respectively, whose values are taken from reference [20].

**Table 2:** Refractive indices and extinction coefficients at the emission wavelength of 459 nm for various materials/layers used in the optical simulation.

Material	n	k
SiO_2_	1.47	0
n-GaN	2.42	4 × 10^−5^
MQW	2.48	2 × 10^−2^
AlGaN	2.35	7 × 10^−4^
p-GaN	2.42	4 × 10^−5^
ITO	2.07	0
n/p metal	2.28	3.06
OC	1.49	0

## Data Availability

The data presented in this study are available on request from the corresponding author.
